# Alterations in the vimentin cytoskeleton in response to single impact load in an *in vitro *model of cartilage damage in the rat

**DOI:** 10.1186/1471-2474-9-94

**Published:** 2008-06-24

**Authors:** Frances MD Henson, Thea A Vincent

**Affiliations:** 1 Department of Veterinary Medicine, University of Cambridge, Madingley Road, Cambridge, CB3 0ES, UK

## Abstract

**Background:**

Animal models have provided much information on molecular and cellular changes in joint disease, particularly OA. However there are limitations to *in vivo* work and single tissue *in vitro* studies can provide more specific information on individual events. The rat is a commonly used laboratory species but at the current time only *in vivo* models of rat OA are available to study. The purpose of this study was to investigate the damage that single impact load (SIL) of 0.16J causes in a rat cartilage *in vitro* model and assess whether this load alters the arrangement of vimentin.

**Methods:**

Rat cartilage was single impact loaded (200 g from 8 cm) and cultured for up to 48 hours (n = 72 joints). Histological changes were measured using a semi-quantitative modified Mankin score. Immunolocalisation was used to identify changes in vimentin distribution.

**Results:**

SIL caused damage in 32/36 cartilage samples. Damage included surface fibrillation, fissures, fragmentation, changes in cellularity and loss of proteoglycan. SIL caused a statistically significant increase in modified Mankin score and chondrocyte clusters over time. SIL caused vimentin disassembly (as evidenced by collapse of vimentin around the nucleus).

**Conclusion:**

This study describes a model of SIL damage to rat cartilage. SIL causes changes in histological/chemical parameters which have been measured using a semi-quantitative modified Mankin score. Single impact load also causes changes in the pattern of vimentin immunoreactivity, indicating vimentin dissassembley. Using a semi-quantitative scoring system the disassembly was shown to be statistically significant in SIL damaged cartilage.

The changes described in this paper suggest that this novel single tissue rat model of joint damage is a possible candidate model to replace *in vivo* models.

## Background

In order to study osteoarthritis (OA) in both man and animals, much use has been made of animal models of pathology to generate consistent, reproducible articular cartilage lesions within a relatively rapid period of time [1]. Whilst animal models of pathology have provided researchers with a wealth of knowledge about events that occur in joint disease, there are drawbacks to their use including the difficulty of isolating different tissue responses to a given insult. This is particularly true in the joint where the synovial membrane, synovial fluid, vasculature, nervous supply, cartilage and underlying subchondral bone all contribute to the end pathology, making elucidation of specific molecular events within one tissue type difficult to isolate and more difficult to interpret. Therefore, in order to study individual tissue responses single tissue experiments are required.

Single impact load (SIL) damage in articular cartilage was first described by Jeffrey et al ([2]). Subsequently a number of papers have investigated the effect of SIL and have shown alterations in matrix loss and synthesis [3], cell volume [4], and an increase in apoptosis in loaded cartilage [5,6]. In addition, a recent paper has described the validation of an *in vitro *SIL model of the initiation of OA-like changes in equine articular cartilage [5]. This work showed that SIL and subsequent culture of cartilage explants caused degenerative changes similar to those observed in OA, including the development of chondrocyte clusters (multinucleate groups of chondrocytes that are considered to be a characteristic histological change of OA). These changes can be quantified and compared, making the *in vitro *SIL model a useful single tissue tool for the elucidation of the early molecular pathways involved in the process leading from mechanical trauma to cartilage degeneration. However, whilst experiments on equine cartilage can provide extremely useful data, equine cartilage can be hard to obtain and standardized and the use of cartilage from a more readily obtainable species, e.g. rat, may be useful in the elucidation of cellular and molecular events that occur with SIL. At the current time there is, to the authors' knowledge, no description in the literature of an *in vitro *model of mechanical joint damage in the rat with experimental models of rat joint disease being surgically induced *in vivo *[7,8]. A rat SIL model of joint damage, would, therefore provide a useful experimental tool.

In order to identify whether joint damage models produce changes similar to clinical disease, quantitative scoring systems have been described [9,10], which are based on structural damage to the cartilage [9]. In addition to identifying such gross structural changes in cartilage, cytoskeletal changes have also been reported in response to mechanical load, specifically changes in vimentin [11].

The chondrocyte cytoskeleton is a three-dimensional network comprised of three types of protein networks: actin microfilaments, tubulin microtubules, and vimentin intermediate filaments. The likely roles of microfilaments are in cell-matrix interactions, cell signaling, differentiation, intracellular transport, control of secretion/endocytosis and in maintaining cell shape [12]. Vimentin intermediate filaments and microtubules form a link between the plasma membrane and the nucleus, with vimentin forming a tighter and finer mesh than microtubules, and these intermediate microfilaments may play a role in the mechanotransduction process [12]. Evidence for a role in the response to load comes from a number of directions, including the response to chondrocyte swelling [11] and chondrocyte deformation experiments [13] and vimentin microfilaments have been shown to contribute to the viscoelastic properties of the chondrocyte [14]. The vimentin knockout mouse has been reported to display no obvious phenotype [15], however, a reduction in stiffness, mechanical stability, motility and directional migration in vimentin-deficient fibroblasts has been described [16]. Of interest in the context of joint disease is the observation that vimentin has also been shown to be altered in naturally occurring OA [17,18].

The aims of this study were (i) to validate a single impact load model of joint damage in rat femoral cartilage *in vitro *and (ii) to identify alterations in the vimentin intermediate filament cytoskeleton in this model over 48 hours in culture.

## Methods

### Tissue samples

Rat cadavers of male Sprague-Dawley rats between 20 and 26 weeks of age were obtained following euthanasia (overdose of barbiturate). After disarticulation of the femur from the acetabulum the proximal femur was dissected free from muscle. The femoral head was then severed from the femoral shaft by sharp excision to a depth of 5 mm from the articular surface using a standardized dissection procedure. The cartilage-bone unit was then placed immediately into sterile phosphate buffered saline (PBS) solution. A total of 84 femoral heads were used (42 rats).

### Impact loading

In order to impact load the rounded cartilage surface evenly a simple cartilage/bone unit loading system was designed. This was basically a lead plate, which had, on its surface an imprint created by the articular surface of the joint. This imprint was made by using a cartilage/bone joint unit obtained as described above from an individual from the same group of rats.

Impact loading was performed by placing the femoral head articular surface downwards on the metal plate. The load was applied to the flat cut bone surface and the cartilage received load that had been transmitted through the bone to the cartilage that was adjacent to the metal plate. Single impact load was performed using a drop tower apparatus as described previously [6]. A weight of 200 g was dropped from a height of 8 cm (corresponding to an impact of 0.16J (calculated from [2]). This impact was chosen after a number of pilot experiments showed that this was the minimum load required, using this experimental design, to induce detectable damage in the cartilage, which could be scored using a modified Mankin system.

### Cartilage damage scoring system

A modified Mankin semi quantitative scoring system [10] was used to quantify changes within the cartilage sections (Table 1). Using this scoring system the maximum score obtainable was 15.

**Table 1:** The components of the modified Mankin scoring system

**Structure**	**Cellularity**	**Matrix staining**	**Tidemark integrity**	**Score**
Smooth surface/normal	Normal arrangement	Normal staining	Normal and intact	0
Roughened surface/single crack or area of deamination	Clustering in superficial layer or loss of cells up to 10%	Slight loss of stain	Disrupted	1
Multiple cracks/moderate delamination	Disorganisation or loss up to 25%	Moderate loss of stain	X	2
Fragmentation in cartilage or severe delamination	Cell rows absent or loss to 50%	Severe loss of stain	X	3
Loss of fragments	Very few cells present	No stain present	X	4
Complete erosion to tidemark	x	X	X	5
Erosion beyond tidemark	X	X	X	6

### Tissue culture and histological staining

Cartilage was washed three times in sterile PBS and incubated in culture medium (DMEM Sigma-Aldrich, UK) supplemented with 200 IU/ml penicillin (Invitrogen, UK), 2.5 μg/ml streptomycin (Invitrogen, UK), 500 μg/ml ascorbic acid (Sigma Aldrich, UK) and 10% fetal calf serum (Invitrogen, UK) at 37 degrees C and 5% CO2. Femoral heads were cultured for 1, 2, 4, 8, 24 and 48 hours. T = 0 was taken to represent samples that had not been cultured, however, this is roughly equivalent to t = 1 minute as this is the approximate time taken to remove the sample from the loading chamber and snap-freeze it. Non impacted femoral heads were cultured as control samples. At each time point the femoral heads were removed, the cartilage removed from the bone and snap frozen. 7 μm sections were obtained and stained with toluidine blue and H&E. All experimental time points were cultured in groups of 6.

### Immunohistochemistry

Frozen sections were fixed in 4% paraformaldehyde for 20 minutes at room temperature and washed in PBS. Immunohistochemistry was performed on cartilage sections using a standard fluorescent secondary antibody detection method. The primary antibodies used were monoclonal mouse anti-pig vimentin (Sigma, UK) at a dilution of 1 in 200, goat polyclonal anti-mouse pERK1/2 (Santa Cruz Biotechnologies) at a dilution of 1 in 100 and goat polyclonal anti-rat ERK (Santa Cruz Biotechnologies) at a dilution of 1 in 100. The secondary antibodies used were FITC-labeled anti-mouse and FITC-labeled anti-goat (Sigma, UK). Sections were counterstained with DAPi to allow identification of the nucleus (Vectorshield + DAPi, Vector Laboratories, UK). Vimentin immunofluorescence was performed at all time points, ERK and pERK at t = 0 and t = 1 hour. ERK and pERK immunofluorescence was performed as a basic indicator of whether or not SIL could cause phosphorylation within this experimental situation. All fluorescent-labeled sections were imaged with a Leitz Laborlux 12 fluorescence microscope using digital image acquisition.

### Vimentin scoring system

**Figure 1:**
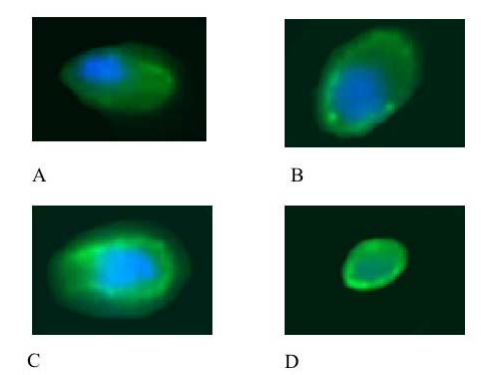
**Photomicrographs to show the representative appearance of the vimentin cytoskeleton within each of the four scoring categories.** Chondrocytes have been stained with monoclonal anti-vimentin antibody and visualized with an immunofluorescent secondary antibody (green). Counter stained with DAPi nuclear stain (blue). Chondrocytes with a normal cytoskeletal appearance i.e. lattice throughout the cell were scored as 0 (A), cells with a slight increase in immunofluorescent intensity of stain around the nucleus were scored as 1 (B), with a moderate intensity around the nucleus 2 (C) and with the stain entirely around the nucleus 3 (D).

The integrity of the vimentin intermediate filaments can be identified by immunofluorescent staining techniques which reveal disassembly as a collapse of the vimentin microfilaments around the nucleus [19]. In order to identify and quantify intermediate filament disassembly in the chondrocytes a scoring system was devised (Figure 1). Chondrocytes with a normal cytoskeletal appearance i.e. lattice throughout the cell were scored as 0 (Figure 1a), cells with a slight increase in immunofluorescent intensity of stain around the nucleus were scored as 1 (Figure 1b), with a moderate intensity around the nucleus 2 (Figure 1c), and with the stain entirely around the nucleus 3 (Figure 1d), In each section a total of 100 chondrocytes were counted throughout the full thickness of the section and the total score for each section derived. A high vimentin score indicates a high level of intermediate filament disassembly. The scoring was performed in a blinded fashion.

### Statistical analysis

Kruskal-Wallis and Mann-Whitney tests were used to identify whether there were significant differences between the control and SIL modified Mankin scores. In order to detect any significant difference between control and SIL vimentin scores the Chi-squared test was used. A result was considered significant when p < 0.05.

## Results

### Histological changes

#### Modified Mankin Score (MMS)

**Figure 2:**
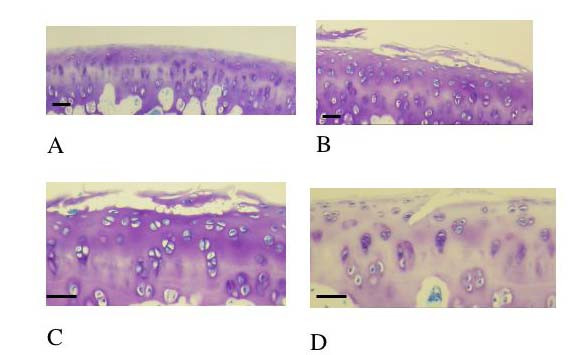
**2a Histological section of cartilage obtained from the distal femur of a rat at 23 weeks of age.** This is a control section i.e. has not received single impact load and it is has not been cultured i.e. represents a control section, T = 0. Stained with toluidine blue. There is no damage to the cartilage; the articular surface is smooth and flat. There are no micro-fractures or fragments and there is no loss of proteoglycan. Scale bar = 10 μm. 2b Histological section of cartilage obtained from the distal femur of a rat at 23 weeks of age. This cartilage has been impacted with a single impact load of 200 g from 8 cm. T = 0. Stained with toluidine blue. There is marked surface damage to the cartilage including lamination. Scale bar = 10 μm. 2c Histological section of cartilage obtained from the distal femur of a rat at 23 weeks of age. This cartilage has been impacted with a single impact load of 200 g from 8 cm. T = 0. Stained with toluidine blue. There is marked surface damage to the cartilage including the formation of a fragment discreet from the parent cartilage. Scale bar = 10 μm. 2d Histological section of cartilage obtained from the distal femur of a rat at 23 weeks of age. This cartilage has been impacted with a single impact load of 200 g from 8 cm. This cartilage has been cultured for 48 hours i.e. T = 48 h. Stained with toluidine blue. There is a fissure at the articular surface and marked proteoglycan loss. Scale bar = 10 μm

**Figure 3:**
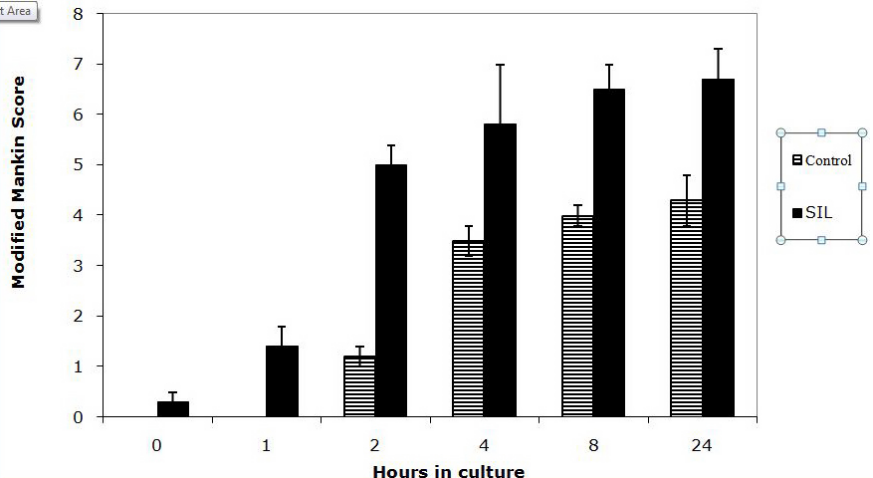
**Graph to show the modified Mankin score in control and single impact loaded (200 g from 8 cm) cartilage over a 48 hour time period.** There is a significant difference between control and single impact loaded cartilage at t = 8, 24 and 48 hours in culture (*).

Following SIL, cartilage damage was seen in 38/42 impacted samples and 4/42 control samples. In non-impacted cartilage no structural damage was noted in 38/42 sections (Figure 2a). In impacted cartilage structural damage included surface damage and delamination of the cartilage surface (Figure 2b), cartilage fragmentation (Figure 2c), fissures (Figure 2d) and loss of proteoglycan content of the cartilage indicated by loss of toluidine blue staining compared to non impacted cartilage (Figure 2d). Using the combined modified MMS a number of differences were shown. Firstly it was shown that, in control sections, the MMS increased with time in culture (Figure 3). This was statistically different from t = 0 at 2, 4, 8, 24 and 48 hours (p < 0.05). Secondly it was shown that, in SIL sections, a similar trend was noted i.e. the MMS increased with time in culture (Figure 3). This was statistically different from t = 0 at 1, 2, 4, 8, 24 and 48 hours. Thirdly it was shown that the MMS was statistically significantly different between control and SIL sections at t = 8, 24 and 48 hours in culture (marked as significant in Figure 3).

#### Proteoglycan loss

In both impacted and control cartilage there was loss of proteoglycan at t = 0 and a subsequent increase over time in culture. There was no statistical difference between control and SIL cartilage (results not shown).

#### Structural and cellular damage

**Figure 4:**
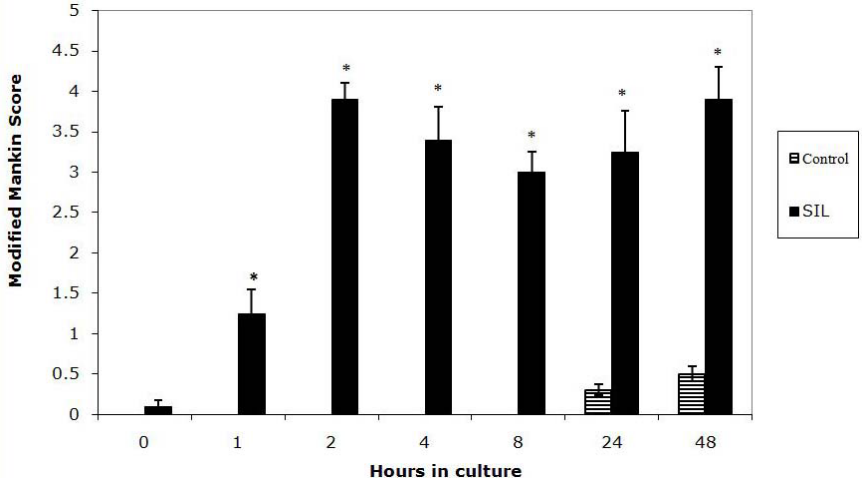
**Graph to show the modified Mankin score minus the proteoglycan component score in control and single impact loaded (200 g from 8 cm) cartilage over a 48 hour time period.** The SIL cartilage is significantly increased relative to the control in all sections.

Having noted that in both control and SIL cartilage there was marked proteoglycan loss during time in culture the MMS minus the proteoglycan score was quantified (Figure 4). This value clearly shows that at t = 1, 2, 4, 8, 24 and 48 hours the structural/cellular changes in the impacted cartilage are statistically increased compared to the control cartilage.

#### Chondrocyte clusters

**Figure 5:**
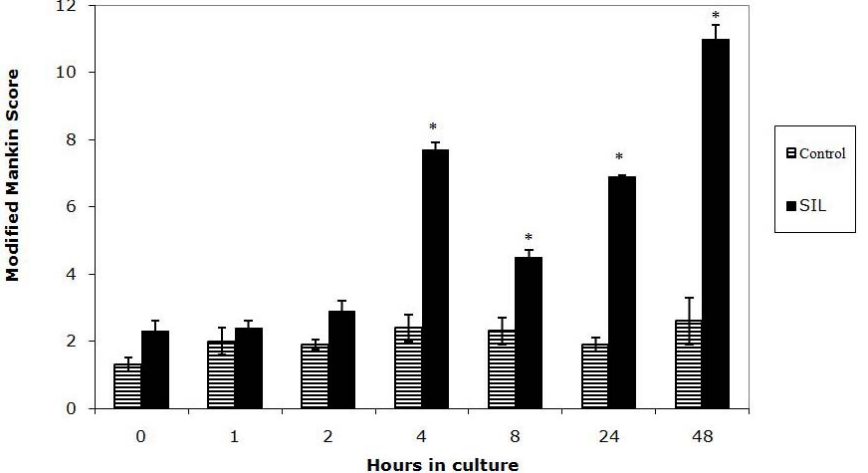
**Graph to show the numbers of chondrocyte clusters in control and single impact loaded (200 g from 8 cm) cartilage over a 48 hour time period.** There is a significant difference between control and single impact loaded cartilage at t= 4, 8, 24 and 48 hours in culture (*).

**Figure 6:**
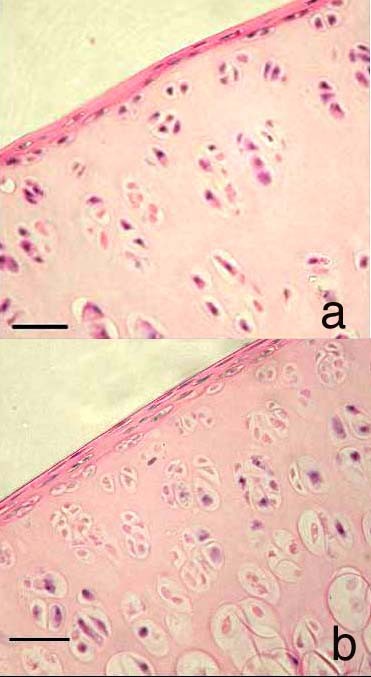
**Histological sections of cartilage obtained from the distal femur of a rat at 23 weeks of age.** 4a. This cartilage has been not been impacted i.e. is a control section but has been cultured for 48 hours. Few chondrocyte clusters (defined as cell groupings of 4+ nuclei, not arranged perpendicular to the joint surface) are present. 4b This cartilage has been subjected to single impact load of 200 g from 8 cm and cultured for 48 hours. There are significantly more chondrocyte clusters in this section than in the control section. Both sections are stained with H&E. Scale bar = 15 μm.

**Figure 7:**
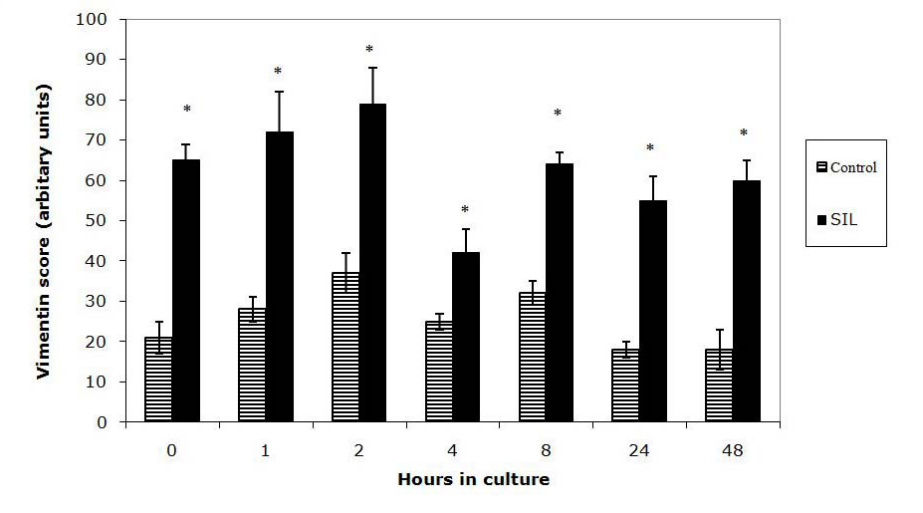
**Graph to show vimentin scoring in cartilage sections in control and single impact loaded (SIL) cartilage over 48 hours in culture.** Vimentin scoring is shown in arbitrary units, with a high score representing increased disassembly of the vimentin. At all time points there is a statistically significant increase in vimentin score in SIL cartilage, * shows statistical significance between the control and the SIL cartilage at each time point.

As part of the MMS the cellularity of the cartilage was scored. This score includes a number of different parameters including loss of cells, disorganisation of the cellular arrangement and the presence of chondrocyte clusters. Chondrocyte clusters were detected in SIL cartilage at all time points. At t = 4, 8, 24, and 48 hours the numbers of chondrocyte clusters in SIL cartilage were statistically significantly increased compared both to control sections and also to SIL cartilage at both t = 0 and t = 1 hours (Figure 5). The histological appearance of the control and SIL cartilage at t = 48 hours is shown in Figures 6 and 7. In 4b there are significantly increased numbers of chondrocyte clusters.

#### Vimentin Immunohistochemistry

In all sections studied immunofluorescence clearly revealed the presence of vimentin intermediate filaments. The staining within each cell was assigned to one of 4 scoring groups, reflecting the degree of disassembly of the cytoskeleton. In control sections no score of 3 was assigned to any chondrocyte, i.e. no cell was seen to have vimentin staining entirely around the nucleus. In contrast, chondrocytes with a score of 3 were seen in single impact loaded sections throughout the culture period, including at time = 0.

**Figure 8:**
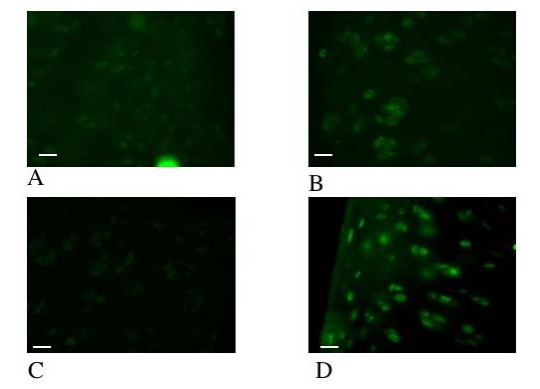
**Photomicrographs of the cartilage from the distal femur of a rat.** The photomicrographs illustrate the immunoreactivity of ERK and pERK within the cartilage. Sections 6a and 6b have been immunohistochemically labeled with anti-ERK antisera and visualized with an immunofluorescent secondary antibody (green), sections 6c and d have been immunohistochemically labeled with anti-pERK antisera and visualized with an immunofluorescent secondary antibody. Sections 6a and 6c represent t= 0 and sections 6 b and d represent t = 1 hour. In Figure 6a (ERK, t = 0) there is immunoreactiviey tht is not ssignificantly different from Figure 6b (ERK, t = 1 hour) i.e. there has been no change in ERK immunoreactivity levels. In contrast in Figure 6c (pERK, t = 0) there is no immunoreactivity i.e. no activation of pERK, whilst in Figure 6d (pERK, t = 1 hour) strong immunoreactivity is seen. Scale bar = 13 μm

In order to compare vimentin disassembly in control and SIL sections the total score for vimentin staining was calculated at each time point. The vimentin score was significantly increased in single impact loaded sections at all time points studied (Figure 8) compared to the control sections i.e. there was statistically significant disassembly in SIL cartilage compared to controls identified by the semi-quantitative scoring system described. The vimentin score peaked at 2 hours and then declined, this cannot be considered statistically significant evidence of re-assembly but does show a trend towards it.

#### ERK/pERK Immunohistochemistry

Immunolocalisation showed that ERK was detectable in the cytoplasm of all chondrocytes at t = 0 and t = 1 hour in control and SIL cartilage. At time = 0 there was no pERK within control sections, but in SIL cartilage strong PERK immunoreactivity was detected in the cytoplasm of all of the chondrocytes within the section (Figure 9). By t = 1 hour pERK was detected in both impacted and control sections.

## Discussion

This study demonstrates that SIL and subsequent culture over 48 hours causes a response in rat femoral head articular cartilage. This response includes significant structural and cellular changes quantified by a MMS system and similar to those reported in *in vivo *OA animal models. SIL also causes disassembly of the vimentin intermediate filament cytoskeleton as detected by immunofluorescence.

Impact load is of interest in joint pathology as it is widely reported that secondary OA is a common sequel to an impact load being applied to a joint [20]. In the experiments described here an impact energy of 0.16J was used to load a cartilage/bone unit with a time to peak load of <0.30 ms [20]. This amount of impact energy is comparable to other published studies of the effect of SIL [4,6,21] and time to peak load within the range of physiologically experienced loads [20].

At the current time SIL studies have been reported in a number of species, with a recent paper describing the validation of an *in vitro *SIL model of the initiation of OA-like changes in equine articular cartilage [5]. In comparison with equine cartilage, rat cartilage is much thinner and contains a growth plate, making the rat SIL model as described here essentially a model of the behaviour of the cartilage/bone subunit, rather than a simple cartilage model as in the horse. Thus direct comparison of this rat SIL model with the equine model should not be made, rather the rat SIL model should be compared to *in vivo *rat models.

The MMS system used in this study clearly demonstrates that SIL and subsequent culture causes a statistically significant increase in histological parameters of cartilage damage. The MMS was statistically significantly increased compared to the control cartilage at 8, 24 and 48 hours in culture. The damage seen in this SIL model is comparable to surgically induced models of OA in the rat, including the loss of proteoglycan and fibrillation described in a medial menisal model of rat OA [8], the surface damage/lamination and fibrillation described in the anterior cruciate ligament transection model of rat OA [22] and the loss of matrix staining, fissures and fibrillation described in a denervation model of rat OA [10]. Thus this model warrants further investigation as a possible candidate for replacing *in vivo *models of joint degeneration.

As previously noted by Salo et al [10], accepting the validity of any scoring system for quantifying joint damage requires the acceptance of the assumption that the severity of OA/joint damage can be determined histologically. Mankin et al [9] showed that the Mankin histological/histochemical score correlated well with measured biochemical parameters in cartilage matrix. In this study we have used an MMS that has been previously published as suitable for assessing changes in rat articular cartilage [10]. All sections were assessed in a blinded fashion and the score used simply to quantify the histological appearance of the joints.

The MMS obtained for control cartilage showed that dissection and subsequent culture also caused an increase in MMS score over 48 hours. In order to examine this observation further the individual components of MMS were examined. It was seen that in both control and SIL cartilage during culture there was marked proteoglycan loss. Loss of proteoglycan from cartilage can occur in a number of situations including, *in vivo*, immobilization of the joint or unloading of the joint and *in vitro *during culture. This loss of proteoglycan from cartilage during culture may well be due to simple diffusion of the macromolecules out of the cartilage although some authors have suggested that may be due to activation of MMPs and/or ADAMs following dissection damage and culture [23]. When the proteoglycan component of the modified Mankin score was subtracted from the structural score, the SIL cartilage was significantly different from the controls at more time points (2, 4, 8, 24 and 48 hours) than previously, indicating that most of the response to dissection is expressed via the loss of proteoglycan.

The MMS is made up of a number of components and of these components chondrocyte clusters were investigated individually. Chondrocyte cluster formation is considered a major characteristic phenotype of OA cartilage [24] and these clusters have been used as markers for OA in gene array experiments [25]. One of the problems with identifying chondrocyte clusters in rat cartilage compared to human cartilage and cartilage from larger mammals is that normal rat cartilage is highly cellular and contains a number of multicellular structures in the mid and deep zones. In normal cartilage, therefore, discriminating genuine chondrocyte clusters can be difficult. In this study we established inclusion criteria for designation of a cluster; clusters were made of cell groupings of 4+ nuclei, not arranged perpendicular to the joint surface. Although we considered this inclusion criteria as reasonably strict it is actually relatively generous, as evidenced by the background levels of approximately 3 clusters per field counted in control cartilage at all time points. However using these inclusion criteria it was demonstrated that chondrocyte clusters are statistically significantly increased in cartilage following single impact load by 4 hours post impact. These rapid response to damage is surprising as chondrocyte doubling times in the rat are approximately 24 hours [26], however the appearance of the cell cluster at 4 hours was observed at this very early time point. The reasons for this apparent response are not known at this current time and warrants further investigation. It is possible that they may represent an alteration of an already occurring cell division event such that the cells do not move apart to form separate lacunae or that they have a mechanical basis – for example microfractures within the cartilage may lead to a passive accumulation of cells within the matrix.

In addition to SIL causing histological changes in the cartilage this work demonstrates that SIL causes statistically significant disassembly of the vimentin intermediate filament cytoskeleton, identified using a semi-quantitative scoring system. This disassembly was identified by vimentin immunolocalisation; detection of vimentin in a cytoplasmic network was considered as normal, whilst collapse of the vimentin around the nucleus was considered characteristic of disassembly [19], as it is in other cell types, e.g. glial cells exposed to acrylamide [27]. In designating nuclear localization of vimentin as disassembly it is appreciated that many reports on the sub-cellular localization of vimentin are produced using confocal microscopy, which provides superior localization of cytoskeletal components. Our studies described here, using standard fluorescent microscopy, may potentially be less absolutely specific about the vimentin localization but the same technique was used for control and impacted sections and so using this technique to compare different experimental conditions appears valid in this experiment.

The vimentin disassembly reported in this study is similar to that reported by other workers; Durrant et al (1999) [11] demonstrated a rapid and reversible response of the chondrocyte cytoskeleton, involving vimentin, to varying mechanical conditions in organ culture. The authors reported a rapid disassembly of the vimentin network at the early stages of culture, which was then subsequently re-established. In the study reported here there was no *statistical *evidence that re-assembly was occurring, however a trend towards re-assembly was noted, which would agree with the published data.

The control of the vimentin network is complicated. Intermediate filaments are in a constant state of rapid turnover of subunits, with a dynamic equilibrium between the polymerised and unpolymerised subunits [28]. Therefore, by tilting the balance of the equilibrium towards depolymerisation, a rapid disassembly of the polymerised form could result. The state of the vimentin appears to be governed by phosphorylation and dephosphorylation events [29], with vimentin disassembly associated with phosphorylation [30]. In *vitro*, the vimentin network has been shown to be a target for a number of kinases, including protein kinase A (PKA) and C (PKC) [31]. In order to identify whether the rapid disassembly of vimentin, seen even at t = 0 (which is roughly equivalent to t = 1 minute as this is the approximate time taken to remove the sample from the loading chamber and snap-freeze it) could be associated with a phosphorylation; immunofluorescence of ERK and pERK was investigated. Phosphorylation of ERK was seen at t = 0 in loaded sections, but not controls, indicating that SIL is, therefore, altering at least 1 phosphorylation pathway at this early time point. It is therefore possible that the vimentin disassembly reported in this study is occurring through a phosphorylation dependent mechanism.

SIL is known to cause a number of changes within cartilage and chondrocytes, including apoptosis [5,6] and alterations in cell volume [4]. Both of these could affect the localisation of vimentin. It has been shown that vimentin, along with other intermediate filament proteins [32,33] is involved in the role of caspases, the key mediators of apoptosis. Caspase proteolysis of vimentin at Asp85 promotes apoptosis by disassembling the vimentin cytoskeleton and amplifying cell death via pro-apoptotic cleavage products [34]. However, caspase cleaved vimentin fragments are described as having a classic appearance of punctate, granular aggregates within the cytoplasm and these aggregates were not a feature of the response to SIL by cartilage in this model. This observation, in our opinion, warrants further investigation.

## Conclusion

In conclusion SIL causes cartilage damage in the rat that is similar to that reported in rat *in vivo *OA models. In addition, SIL causes disassembly of the vimetin cytoskeleton, possibly via phosphorylation of the vimentin. The role of this disassembly is not known, but it may be an important part of the chondrocyte mechanotransduction pathway.

## Competing interests

The authors declare that they have no competing interests.

## Authors' contributions

FMDH designed the study and drafted the manuscript, TAV performed the experimental work and the statistical analysis.

## Pre-publication history

The pre-publication history for this paper can be accessed here:


